# Daily milk intake improves vitamin B-12 status in young vegetarian Indians: an intervention trial

**DOI:** 10.1186/1475-2891-12-136

**Published:** 2013-10-09

**Authors:** Sadanand Naik, Vijayshri Bhide, Ashish Babhulkar, Namita Mahalle, Sonali Parab, Ravi Thakre, Mohan Kulkarni

**Affiliations:** 1 Department of Clinical Biochemistry & Orthopedics, Deenanath Mangeshkar Hospital & Research Centre, Erandvane, Pune, India; 2 Department of Endocrinology, K.E.M. Hospital, Rasta Peth, Pune, India; 3 Department of Biochemistry, Pune University, Ganesh Khind Road, Pune, India

**Keywords:** Milk, Vitamin B-12 status, Holotranscobalamin II, Vitamin B-12, Folate, Total homocysteine, Cobalamin

## Abstract

**Background:**

Asymptomatic Indian lacto vegetarians, who make up more than half of the Indian population in different geographic regions, have distinctly low vitamin B-12 concentrations than non- vegetarians. Vegetarians consume milk but it seems that the amount is not enough to improve vitamin B-12 status or vitamin B-12 concentration in milk itself may be low. The aim of this study was to determine if daily milk consumption can improve vitamin B-12 status.

**Methods:**

Fifteen male and 36 female, young healthy post-graduate volunteers participated. Blood from ten participants (4 males and 6 females) was collected (day-1). They continued their regular diet for next fourteen days and on day-15, blood of all 51 participants was collected, plasma vitamin B-12 concentration was measured and were divided into two groups; Normal (vitamin B-12 >148 pmol/L, n = 22) and Vitamin B-12 deficient (<148 pmol/L, n = 29), the remaining plasma was stored. All participants consumed 600 ml. of non-enriched buffalo milk (200 × 3) during the day along with their usual diet. Next day blood was collected for plasma holotranscobalamin II measurement. Subjects from deficient group continued to drink 400 ml of milk daily for next 14 days and blood was collected on day-30. Plasma holotranscoabalamin II (day-1, 15, 16, 30), vitamin B-12, folate, total homocysteine, creatinine and hematoloical parameters (day-1, 15, 30), and milk vitamin B-12 concentrations (day-15, 16, 30) were measured.

**Results:**

Fifty seven per cent of the participants were vitamin B-12 deficient and 65% were hyperhomocysteinemic. No significant difference in biomarkers was observed when there was no intervention. Plasma holotranscobalamin II concentration increased from 19.6 to 22.27 pmol/L (p < 0.0001) 24 hrs after milk load in the whole group. Plasma vitamin B-12 increased from 92.5 to 122 pmol/L and tHcy concentrations decreased from 31.9 to 24.9 μ mol/L (p < 0.0001 for both) 14 days after regular milk intake in vitamin B-12 deficient subjects.

**Conclusions:**

Regular intake of milk improved vitamin B-12 status of vitamin B-12 deficient vegetarians indicating a potential dietary strategy to improve the vitamin status.

## Background

Dietary reference intake of vitamin B-12 was defined as the amount required to prevent the overt vitamin B-12 deficiency that causes megaloblastic anemia and to maintain plasma concentrations above 148 pmol/L [1]. The recommended dietary allowance (RDA) of vitamin B-12 for adults in India is 1.0 μ g/day [2], whereas in USA it is 2.4 μ g/day [3]. Vitamin B-12 intake by Indians derives from natural food products, i.e. dairy products viz. milk, yogurt, and cheese/butter, tea/coffee with milk or from vitamin B-12 supplements. The relation between dietary intake and vitamin B-12 status has been studied in different populations with conflicting results. Howard et al [4] concluded that high frequency of mildly abnormal cobalamin status in the elderly was not due to poor intake of cobalamin, whereas, Framingham Offspring [5] and Danish Study [6] showed significant associations between dietary intake of vitamin B-12 and plasma concentrations. In a large population-based study, Vogiatzoglou et al [7] found a significant association between total daily dietary intake of vitamin B-12 and plasma vitamin B-12 concentrations. They further stated that daily dietary intake of 6-10 μg of vitamin B-12 ensured the maximum plasma vitamin B-12 concentration in persons with adequate vitamin B-12 absorption suggesting that the current RDA of 2.4 μ g/day is also insufficient. The Framingham Offspring Study [5] first observed that vitamin B-12 from milk was better absorbed than meat. Wokes et al [8] systematically compared a group of US, Dutch and British vegans with non-vegetarians from those same countries and found that many of the vegans had significantly lower vitamin B-12 concentrations than did non-vegetarians. Dhopeshwarkar et al [9] showed that asymptomatic Indian lacto vegetarians, who make up more than half of the Indian population, have distinctly low vitamin B-12 concentrations than non-vegetarians which was confirmed by studies from different geographic regions of India [10-14]. Two main etiologic factors play a role in developing vitamin B-12 deficiency; inadequate dietary intake and/or vitamin B-12 mal-absorption.

Bhat et al [15] have used the rise in plasma holotranscobalamin II ( holo-TC) concentrations with a smaller dose of cyanocobalamin (6 μ g) in vitamin B-12 deficient Indians as a marker of absorption. The increase in plasma holo-TC concentration after vitamin B-12 load provided experimental evidence that the increase was due to recent vitamin B-12 absorption. The deficiency of vitamin B-12 has been associated with diverse disorders throughout the life span, from birth defects such as shunted growth, neural tube defects, anemia to hyperhomocysteinemia and neuro-cognitive disorders in adulthood [16-18]. Recently, low plasma vitamin B-12 concentrations in pregnancy have been shown to be associated with diminished neuro-cognitive performance in offspring [19]. Several morbidities may be associated with low-normal vitamin B-12 status (< 148 pmol/L). Therefore, it is important to establish whether dietary intervention by the available natural source can improve vitamin B-12 status in Indian vegetarian population and protect from diverse disorders.

The option of increasing overall vitamin B-12 status of deficient population is either fortification or targeted dietary recommendation. The present study measured the effects of supplementation with 600 ml of skimmed buffalo milk not enriched with vitamin B-12, during the day on circulating holo-TC concentration. It also measured the supplementary effects with 400 ml of non-enriched milk daily for 14 days on vitamin B-12 status by measuring the changes in plasma holo-TC, vitamin B-12 and total homocysteine (Hcy) concentrations.

### Subjects and methods

#### *Participants*

Young, healthy and post graduate students and staff members of Deenanath Mangeshkar Hospital were invited to visit the laboratory. They were told about the high prevalence of vitamin B-12 deficiency in Indian vegetarians and requested to participate in the intervention study. Pregnant women, non-vegetarians and lactose intolerant subjects were requested not to participate. Fifty one volunteers consented to participate in the study (15 males and 36 females, day-0). They had no clinical symptoms of vitamin B-12 deficiency and were not taking vitamin B-12 supplementation or any drugs known to influence vitamin B-12 absorption. All were vegetarians, non-smokers and none consumed alcohol. Their milk intake was not more than 2 cups (120-140 mL) per day. After recruitment, each participant was instructed to report to the laboratory every day and give information about their regular timely milk intake.

They were also instructed to not to change their life style during the study period.

### Study design

#### *Intra-individual variation*

Ten participants were selected for evaluating intra-individual variation in the measurement of plasma vitamin B-12, holo-TC, folate and tHcy concentrations. They were instructed to not to change their life-style during the study period. Their fasting blood was collected in EDTA vacutainer on day-1 and day-15.

#### *24-hrs. milk load test*

**Figure 1:**
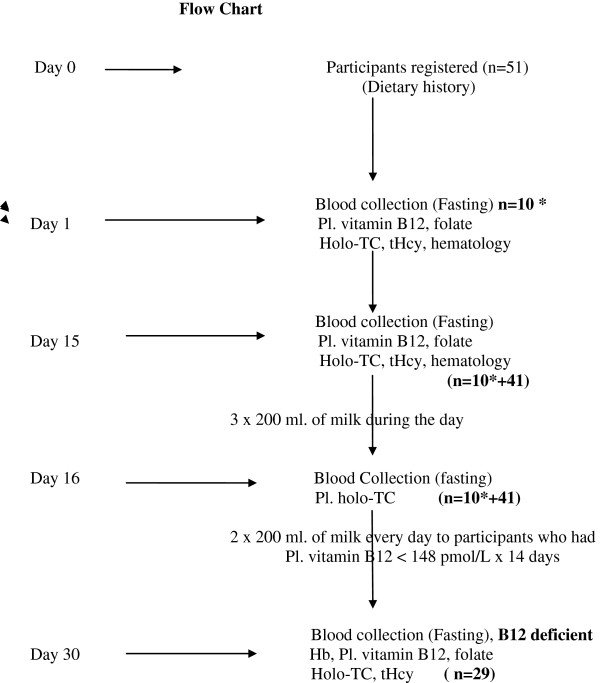
**Flow chart: 51 Participants were registered on day-0.** Fasting blood was collected of 10 participants on day-1*. They were instructed to continue their usual diet for next 14 days. On day-15 fasting blood was collected of all 51 participants. They consumed 3 × 200 ml of milk during the day. On day-16 fasting blood was collected. They were divided into normal and vitamin B-12 deficient groups as per pl. vitamin B-12 concentrations. Deficient participants consumed 2 × 200 ml. milk every day for next 14 days and their fasting blood was collected on day-30. Dietary history was taken on day 1, 15, 30. Participant’s milk drinking time was in the morning after breakfast, 4 p.m. and at night after dinner.

Fasting blood of other 41 healthy participants was collected in EDTA vacutainers on day-15. A portion of blood was processed for hematological parameters within 30 minutes of collection and the rest was centrifuged at 1500 rpm for 20 minutes and the plasma was separated and distributed in four storage vials and stored at -20°C. All of them (51 participants) were instructed to drink 3×200 ml of milk along with their usual diet during the day (in the morning after breakfast, evening at 4 p.m. and at night after dinner) and come to the Laboratory next morning-9 am. On day-16, 10 ml. of fasting blood was collected and the separated plasma was stored as above (Figure 1: Flow sheet).

#### *Effect of daily milk intake on vitamin B-12 status*

Plasma (day-15) vitamin B-12 was measured and the participants were divided into two groups; normal group (plasma vitamin B-12 > 148 pmol/L, n = 22) and the deficient group (<148 pmol/L, n = 29). The participants from the deficient group were instructed to drink 2×200 ml of milk (in the morning after breakfast and after dinner) every day along with their usual diet for the next 14 days and come to the laboratory on day-30. 10 ml. of fasting blood was collected and processed as above. None of the participants neither failed to drink milk nor omitted (reported to the investigator on day-16, day-17 and then every day morning till day-30). There was 100% compliance in milk consumption from the participants. They provided the sample (about 6 ml) of milk consumed for measuring vitamin B-12 concentrations on day 15, 16 and 30 (Figure 1. Flow sheet).

### Experimental procedure

Hematological parameters were measured on 5 part differential cell counter (Sysmex, Cobe, Japan). Plasma holo-TC concentration was measured using microparticle enzyme immunoassay [20]. Microparticle enzyme intrinsic factor assay was used for the quantitative determination of plasma vitamin B-12. Plasma folate and tHcy were measured by fluorescent polarization immune assay technique. Milk samples were incubated with denaturant (0.8 N sodium hydroxide solution with 0.005% potassium cyanide) in 9:1 proportion for 15 minutes at 56°C, centrifuged and processed for vitamin B-12 estimation after diluting with saline (1:10) as mentioned for plasma. All the milk samples were processed for vitamin B-12 measurements on the same day of collection. These biomarkers were measured on Axsym System (Abbott Laboratories, IL, USA). Plasma creatinine was measured on Daytona (Randox, U.K.) using alkaline picrate method [21]. The intra- and inter-assay coefficients of variations were less than 6.2% for hematological and biochemical (vitamin B-12, folate, holo TC, creatinine and tHcy) parameters.

### Dietary intake data

The participants were examined for gross clinical signs of protein-energy under-nutrition and vitamin deficiencies (vitamin A, B-complex, C and D). Staff enquired about upper or lower gastro intestinal disease symptoms and also about milk intolerance. They were interviewed regarding eating habits and health status before enrolling. They consumed their habitual diet throughout the study. Their dietary history (24 hr recall) was taken by trained and experienced personnel at each visit (day-1, day-15, day-30). 24 hr. diet recall questionnaire was administered to record the portion size and quantity of consumption of different foods which were rich in micronutrients at both visits [22]. Foods rich in vitamin B-12 and folate were selected and grouped into four groups; milk and milk containing beverages, tea/coffee with milk, green leafy vegetables and other vegetables. Vitamin B-12 and folate intake were calculated using Dietsoft [23].

### Ethics statement

The study was approved by the ethical committee of Deenanath Mangeshkar Hospital & Research Centre, Pune. Written informed consent was obtained from all participants before enrolling into the study.

### Dietary supplement

Vitamin B-12 concentrations in the milk samples from 12 different sources of non-enriched of milk (buffalo) supply being consumed by the Pune population were measured on three occasions (day-15, 16, 30).

### Definitions

Folate and vitamin B-12 deficiency were defined as plasma concentrations <2 ng/ml [24] and < 148 pmol/L [24] respectively, hyperhomocysteinemia as plasma tHcy concentrations >15 μ mol/L [25], low holo-TC concentration as <35 pmol/L [26], anemia as a hemoglobin concentration <120 g/L in females and <130 g/L in males and macrocytosis as mean corpuscular volume > 100 fL [27].

### Statistical analysis

Intra individual variation was calculated from the estimation of variance by ANOVA from the measurements of the analytes from the two samples obtained from day-1 and day-15 before intervention.

The data is presented as median with 25^th^ and 75^th^ percentiles. Differences between groups were tested using Wilcoxon’s Signed rank matched pair test, a non parametric test, for paired analysis. Association was measured using Pearson Correlation Coefficient. Relative percentage change was calculated with respect to pre-supplementation value. The analysis was performed using SPSS (version 12.0, Chicago, USA).

## Results

The vitamin B-12 concentration in milk supplied by the sources of Pune supply is shown in Table 1; ranges from 2.50 to 3.85 μ g/L. There was no significant variation in vitamin B-12 content of the milk at different times. Fifty one subjects (15 male and 36 female) participated in the study. Evaluating 24-hr recall questionnaire, male subjects were consuming 1.1 μ g of vitamin B-12 per day while females 1.35 μg per day. Main dietary sources of vitamin B-12 are listed in Table 2. Milk represented the predominant source of vitamin B-12 followed by yogurt. Tea/coffee with milk also contributed to some extent in these participants.

**Table 1:** Vitamin B-12 concentration in the milk (Sources of Pune supply)

**Milk source**	**Vitamin B-12 (μ g/L)**		
	**Day-15**	**Day-16**	**Day-30**
Chitale (n = 30)	3.85(3.7,4.1)	4.0 (3.7,4.2)	3.9 (3.7,4.0) n = 8
Rajhans (n = 3)	2.90, 3.1,2.9	2.8,2.8, 2.9	3.0, 3.2, 3.3
Katraj dairy (n = 3)	3.0, 3.2, 3.0	3.2, 3.0, 3.1	3.0, 2.8, 3.2
Godavari (n = 3)	2.95, 3.0. 3.1	3.3, 3.2, 3.0	3.3, 3.0, 3.3
Gowardhan (n = 1)	2.90	3.0	3.2
Bhaiya Rasta Peth (n = 1)	2.50	2.8	2.6
Sane dairy (n = 3)	2.7, 2.90, 3.0	3.1, 3.3, 3.0	3.2
Swarkar dairy (n = 3)	2.80, 3.1, 3.0	3.0, 3.3, 3.1	2.8, 3.2, 3.1
Amol milk (n = 1)	3.05	3.0	3.2
Baiya – Mundhwa (n = 3)	2.60, 2.8, 2.8	3.0, 2.8,3.0	3.0, 3.1, 2.8
Aarey milk (n = 1)	3.2	3.1	3.2
Someshwar dairy (n = 1)	.8	3.0	3.0

The vitamin B-12 contents were similar throughout the study.

Median (25^th^, 75^th^ centile).

n = number of participants consuming milk. Values are the vitamin B-12 concn. in the milk.

**Table 2:** Contribution of vitamin B-12 from different food groups in the participants

**Food intake**	**Quantity/d**	**Vitamin B-12 intake (μ g/d)**
Dairy products		
Tea/coffee - with milk (g/d)	40 (30, 55)	0.18 (0.10, 0.22)
Milk (ml/d)	140 (120, 160)	0.56 (0.5, 1.03)
Yogurt (g/d)	70 (45, 120)	0.15 (0.06, 0.3)
Cheese/butter (g/d)	10 (9, 20)	0.1 (0.08, 0.18)

Median (25^th^ centile, 75th centile).

Vitamin B-12 intake was lower in deficient group than the normal group (p = 0.02). Vitamin B-12 intake was directly related to plasma vitamin B-12 and holo-TC (r = 0.520 and 0.270 respectively, p = 0.001 for both) and inversely to tHcy concentrations (r = -0.430, p = 0.001). The relation was similar in both sexes. There was no folate deficiency and the plasma folate concentrations were similar in both the groups. Calorie, protein and fat intake were also similar and within the limits of Indian Council of Medical Research recommendation [28] (Table 3).

**Table 3:** Dietary intake (24-hr recall) and basic biochemistry of the participants (normal & deficient)

	**All (n = 51)**	**Normal (n = 22)**	**Deficient (n = 29)**	**p value**
Age (years)	27.6(26,30)	29.0(27 – 31.0)	30.6 (28.5-32)	ns
Male/female	14/37	4/18	10/19	
Energy intake (calorie)/d	2240 (2080,2280)	2390 (2220,2480)	2200 (2080,2270)	ns
Protein intake (g)/d	62 (52,80)	58 (50,82)	64 (55,80)	ns
Fat (g/d)	45 (38,50)	44 (35,50)	43 (37,55)	ns
Vitamin B-12 intake (μ g/day)	1.20	1.65	0.65	<0.02
	(0.75,1.50)	(1.4,1.85)	(0.50,0.90)	
Folate intake (μ g/day)	355 (320,400)	350.6 (310.4,415.4)	355.6 (318.2,425.4)	ns
Hemoglobin (g/L)	127 (117,136)	124 (114,130)	128 (123,135)	ns
Anemia%	14	14	14	
Mean corpuscular volume (fL)	84.0 (81.0,90.5)	80.5 (75.0,85.0)	84.3 (82.2,90.4)	ns
Plasma creatinine (mg/dL)	1.0 (0.9,1.1)	0.9 (0.8,1.1)	1.0 (0.9,1.1)	ns
Plasma vitamin B-12* (pmol/L)	130 (98,217)	244 (187,306)	92.8 (71,117)	<0.0001
Plasma holo-TC (pmol/L)*	19.6 (13.1-31.1)	27.7 (19.8,42.5)	14.4 (10.82,19.75)	<0.0001
Plasma folate (ng/mL)	6.24 (4.2,14.90)	6.6 (4.0,15.2)	5.6 (4.6,12.8)	ns
Plasma tHcy ** (μ mol/L)	21.1 (12.7,32.7)	11.9 (10.4,15.0)	31.9 (22.6,54.0)	<0.001

Median(25^th^, 75^th^ centile) ns-non significant.

p- difference between normal group & deficient group.

* Vitamin B-12 intake is directly associated with pl. vitamin B-12 and holo-TC concentrations(r = 0.52 & 0.27, p < 0.001 for both) adjusting for sex.

** vitamin B-12 intake is inversely associated with pl. tHcy concentrations(r = - 0.43, p < 0.001).

The intra individual variation for holo-TC, vitamin B-12, folate and tHcy were less than 6.2% for all, when there was no intervention in their routine life style (Table 4). There was no significant difference in vitamin B-12 intake at different times (Table 1).

**Table 4:** Effect of regular milk intake (14 days) on biomarkers

**Milk intake 400 ml**	**Deficient group**			**No intervention group n = 10 (ns- for all)**	
**Every day**	**n = 29**		**p value**		
	**Day-15**	**Day-30**		**Day-1**	**Day-15**
Hemoglobin (g/L)	128(123,135)	131(125,140)	ns	130(120,140)	127(117,136)
Mean corpuscular volume (fL)	84.3 (82.2,90.4)	86.2 (83.5,91.0)	ns	86.4(82.0,92.0)	85.4(82.2,90.5)
Creatinine (mg/dL)	1.0 (0.9,1.1)	0.9(0.85,1.1)	ns	1.0(0.95,1.2)	1.0(0.9,1.1)
Pl. vitamin B-12 (pmol/L)	92.8 (71.0,117.6)	122.1 (90.6,141.3)	<0.0001	115.5 (97.7, 177.6)	113.9 (102.1, 173.2)
Pl. folate (ng/mL)	5.6 (4.6, 12.8)	5.7 (4.6, 11.9)	ns	6.1 (4.4, 13.8)	5.9 (4.2, 14.9)
Pl. holo-TC (pmol/L)	14.4 (10.82, 19.75)	19.2 14.95,24.6)	<0.0001	19.2(10.2,23.5)	19.5(11.1,22.9)
Pl. tHcy (μ mol/L)	31.9 (22.6,54.0)	24.9 (18.54,41.45)	<0.0001	22.85 (20.5,35.7)	23.9 (20.7,31.7)

p-values by Wilcoxon’s Signed rank test, a non-parametric test for paired analysis ns- not significant. Median (25th, 75th %le).

Hemoglobin, mean corpuscular volume, plasma creatinine and tHcy were significantly higher in males (p < 0.001 for all). Plasma vitamin B-12 and holo-TC concentrations were significantly higher in females (p < 0.001 for both).

**Figure 2:**
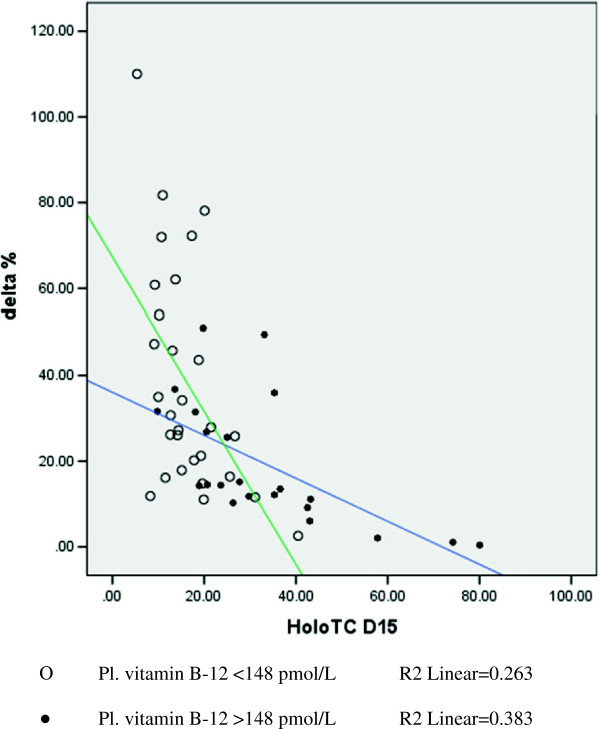
Percentage increase in plasma holo-TC concentration in participants 24hr after 600 ml of milk load from base line (day-15).

Table 5 compares the extent of increase in plasma holo-TC concentrations 24 hrs after the milk load (3 × 200 ml). None of the participants complained of any sort of discomfort and none suffered from diarrhea. The increase in plasma holo-TC concentration 24 hrs after 600 ml milk load, the median (range) increase as (%age and absolute), was 25.8 (0-110%) and 4.2 (0-16.4) pmol/L (p < 0.01). 1/4^th^ of the participants showed less than 3.0 pmol/L (12.5%) increase, half showed more than 26% and one fourth showed more than 43% increase. The participants showing less than 12.5% increase can be labeled as poor absorbers. Percentage increase in plasma holo-TC concentration was independent of basal holo-TC concentration (Figure 2). The increase in plasma holo-TC concentration 15 days after 400 ml milk intake every day, the median (range) increase as (%age and absolute), was 35.1 (5.1-201%) and 5.0 (1-25.6 pmol/L). Similarly the decrease in plasma tHcy concentration was 9.7(0.7-49.3%) and 6.9 (0.5-17 μmol/L) respectively in vitamin B-12 deficient participants (p < 0.0001 for both). The significance was similar in both sexes. We also divided the participants by the observed median holo-TC concentration (22.7 pmol/L) and by <35 > pmol/L (reference cutoff). Both interpret the significant increase in plasma holo-TC 24 hr after milk load (Tables 6, 7).

**Table 5:** Effect of milk load (600 ml) on plasma holo-TC concentrations

	**All (n = 51)**		**Normal group (n = 22)**		**Deficient group (n = 29)**	
	**Day-15**	Day-16 *	**Day-15**	**Day-16 N.S.**	**Day-15**	Day-16 ***
Plasma holo-TC concn.	19.6	22.27	27.7	31.3	14.4	18.3
(pmol/L)	(13.1,31.1)	(16.6,35.8)	(19.8,42.5)	(21.6,46.1)	(10.8,19.7)	(13.7,25.2)

Values are Median (25^th^, 75^th^ centile). p-values by Wilcoxon’s Signed rank test, a non- parametric test for paired analysis (* p <0.01, *** p < 0.0001, N.S.- not significant).

**Table 6:** Plasma holo-TC increase considering the cutoff for holo-TC as 35 pmol/L(reference cutoff)

	**All participants n = 51**		**Pl. holo-TC > 35pmol/L (med vit B-12 267 pmol/L) n = 11**		**Pl. holo-TC < 35pmol/L (med vit B-12 155 pmol/L) n = 40**	
Pl. holo-TC pmol/L	Day-15	Day-16 P = 0.001	Day-15	Day-16 P = 0.013	Day-15	Day-16 P < 0.0001
	19.6 (13.1,31.1)	22.27 (16.6,35.8)	42.7 (36.2,61.9)	47.2 (44.1,60.7)	18.4 (16.6,22.0)	20.45 (15.6,29.1)

Median (25^th^, 75^th^ centile).

p-compared to day-15 concentration.

**Table 7:** Plasma holo-TC increase considering the cutoff for holo-TC as median obtained in the present study (22.7 pmol/L)

**Pl. holo-TC > 22.7pmol/L (med vit B-12 280 pmol/L) n = 19**		**Pl. holo-TC < 22.7pmol/L (med vit B-12 139 pmol/L) n = 32**	
Day-15	Day-16 P = 0.002	Day-15	Day-16 P < 0.0001
36.5 (33.1,43.1)	45.5 (35.8,50.6)	14.7 (11.1,19.2)	18.5 (14.1,23.1)

median (25th, 75th centile).

p-compared to day-a5 concentration.

Holo-TC: vitamin B-12 ratio. [Median (25^th^, 75%le)] on day-15 of all participants was 0.11(0.08,0.16). Normal participants showed 0.08 (0.07,0.12) and deficient showed 0.14 (0.0.09,0.17) on day-15 and 0.13 (0.010,0.18) on day 30. Deficient participants showed increased plasma holo-TC but not significant.

Plasma holo-TC concentration did not rise after milk load in 4 normal and 2 deficient subjects. The basal plasma holo-TC concentrations were more than 45 pmol/L and tHcy less than 12.5 μ mol/L in the normal subjects in whom there was no rise in holo-TC concentrations. The two vitamin B-12 deficient participants did not respond to the 24 hr milk intake. However, their plasma vitamin B-12 (50, 105 pmol/L) increased to (60,116), holo-TC (13.1, 19.4 pmol/L) increased to (14.9, 21.5) and tHcy (58.7, 32.8 μmol/L) decreased to (53.6, 28.1) respectively after 14 days of regular milk intake.

There was no significant difference in hemoglobin, MCV, plasma creatinine and folate concentrations in vitamin B-12 deficient participants 14 days after regular intake of milk, whereas, the increase in plasma vitamin B-12, holo-TC and the decrease in plasma tHcy concentration was significant (p < 0.0001 for all), Table 4. There was no gender difference in the improvement of vitamin B-12 status.

Basal hemoglobin was not associated with circulating vitamin B-12 concentration. However, mean corpuscular volume was inversely related to plasma vitamin B-12. Intake of calorie, carbohydrates, fats and proteins did not change the strength of the association of milk load with holo-TC, vitamin B-12 and tHcy concentrations.

## Discussion

The present study is to determine the beneficial effect of regular consumption of adequate milk on vitamin B-12 status in predominantly young vitamin B-12 deficient vegetarian Indians who had no folate deficiency. Milk was only found to be a significant contributor to vitamin B-12 intake in these participants.

Fifty seven percent of the participants had vitamin B-12 deficiency and 65% had hyperhomocysteinemia, 84% had low plasma holo-TC (<35 pmol/L) concentrations. However, none had any clinical (neurological/anemia) symptoms of vitamin B-12 deficiency. They could be termed as subjects with Sub Clinical Cobalamin deficiency. One half of the participants with normal vitamin B-12 status (plasma vitamin B-12 >148 pmol/L and tHcy <15 μ mol/L) had lower plasma holo-TC concentrations (median 27.7 pmol/L) than normal white Caucasians [5,6,29,30]. The participants were consuming less than 1.4 μ g vitamin B-12 in daily diet. There was a significant association between dietary intake of vitamin B-12 and plasma vitamin B-12 and holo-TC concentrations which is in agreement with other studies [5,7,29].

Holo-TC has a short half-life [31,32] and is therefore proposed to reflect recent vitamin B-12 absorption. Once in circulation, holo-TC is taken up into cells within minutes [33,34]. Until the cells are saturated with holo-TC, most of it is taken up so quickly that no large changes are observed initially in blood. Significant changes in the plasma holo-TC concentrations can be measured when the intake of vitamin B-12 is sufficient to saturate the cells with vitamin B-12. A dose dependent Holo-TC rise during 24 hrs following vitamin B-12 administration suggested that the levels are influenced by recent absorption [15]. Plasma holo-TC concentration significantly increased (0-120%, p = 0.0001) in vitamin B-12 deficient participants 24 hrs. following the milk load (Figure 2). Bhat et al [15] found a significant increase in plasma holo-TC concentration (0-150%) 24 hrs after loading with 6 μg of cyanocobalamin in vitamin B-12 deficient subjects while Bor et al [35] found an increase (0-108%) in normal subjects after 27 μg load. Bor et al [35] and Castel-Roberts et al [36] reported that measuring the rise in plasma holo-TC concentration 24 hrs after vitamin B-12 load is better to assess recent intestinal absorption than measuring plasma vitamin B-12 alone. One fourth of the participants showed <12.5% increase in holo-TC concentration after milk load. They may have mal-absorption or the ingested vitamin B-12 may have been used to saturate the cells. However, their plasma tHcy was reduced in vitamin B-12 deficient participants. These subjects need further evaluation for their gastrointestinal absorption status. Whereas, the participants with normal plasma B-12 concentration who did not show any increase in plasma holo-TC concentrations had basal level of >45 pmol/L with <12.5 μ mol/L of plasma tHcy which implies that plasma holo-TC concentration reaches a plateau when basal plasma holo-TC concentration exceeds 45 pmol/L in vitamin B-12 deficients, similar to the observation by Bhat et al [15] who reported that plasma Holo-TC concentration increased from 10.6 pmol/L to 24.9 in adult Indian males and 30.5 pmol/L in females after ingesting 6 μ g of cyanocobalamin and 7.7 to 45.7 in males and 9.7 to 47.5 pmol/L in females after 30 μ g of cyanocobalamin ingestion.

Regular consumption of milk for 14 days increased the circulating concentration of vitamin B-12, holo-TC and decreased tHcy concentration in vitamin B-12 deficient participants indicating efficient metabolic effects (Table 4).

Carmel [37] categorized available biomarkers as those that directly measured plasma vitamin B-12 and those that measured metabolites that accumulated with inadequate amounts of vitamin B-12. Serum holo-TC and vitamin B-12 measured circulating vitamin B-12 concentrations. These two therefore reflected the broad vitamin B-12 status from high risk of severe deficiency to adequacy. Miller et al [38] stated that holo-TC and total vitamin B-12 have equal diagnostic accuracy in screening for metabolic vitamin B-12 deficiency. Measurement of both holo-TC and total vitamin B-12 provided a better screen for vitamin B-12 deficiency than either assay alone.

According to Green [39], low vitamin B-12 status was indicated by being below the lower the reference range (for vitamin B-12 < 148 pmol/L; for holo TC, <35 pmol/L), whereas for indirect measures of metabolites (methylmalonic acid or homocysteine), low vitamin B-12 status measures would be indicated by a level above the upper limit of the reference range (for methylmalonic acid, >260 nmol/L; for homocysteine, >12 μmol/L). The markers he mentioned were homocysteine for detection of either vitamin B-12 or folate deficiency and methylmalonic acid for vitamin B-12 deficiency only.

However, Valente et al [40] suggested a diagnostic strategy using holo-TC as the front-line test. The cutoffs for deficiency were defined as 20 pmol/L for holo-TC and 123 pmol/L for serum vitamin B-12 after studying employees and medical students of a local hospital at Dundee, UK. We defined the participants as vitamin B-12 deficient who showed <148 pmol/L of plasma vitamin B-12 concentration.

Yajnik et al [41] observed that the habitual intake of green leafy vegetables or other vegetables did not affect plasma vitamin B-12 or tHcy concentrations. They [41] have reported that the oral vitamin B-12 supplementation reduced plasma tHcy concentration in first two weeks but did not achieve normal plasma tHcy concentrations even after 6 wks. Deshmukh et al [42] studied the effects of physiological doses of oral vitamin B-12 on plasma tHcy and observed that the reduction was 5.5 and 6.8 μ mol/L after 4 and 12 months of 2 μ g supplementation per day respectively, while supplementation of 10 ug per day for 6 and 12 months reduced plasma tHcy concentration by 5.6 and 6.9 μ mol/L respectively. There was no advantage in supplementing with higher dose. It has been observed in healthy adults that about 50% of a 1-μg dose of vitamin B-12 is absorbed from food, while only 20% of a 5 μ g dose, and 5% of a 25 μ g dose is absorbed [43]. Therefore, intervention with at least 1.0 to 1.54 μ g of additional vitamin B-12 available from milk every day producing beneficial metabolic effect, increasing plasma vitamin B-12 and reducing tHcy, is advocated.

The novelty of the present study is that it used milk supplementation to measure both absorption capacity and efficiency to reduce plasma tHcy levels. Bor et al [35], von Castel-Roberts et al [36] and Bhat et al [15], used free cyanocobalamin to assess intestinal vitamin B-12 absorption while Deshmukh et al [43] used free cyanocobalamin for lowering plasma homocysteine levels. Although cyanocobalamin increases circulating levels of cobalamin, its ability to increase tissue levels of the active form of vitamin B-12 can be limited in a large number of subclinical and clinical conditions. The activation of cyanocobalamin to either adenosyl cobalamin or methyl cobalamin does not occur instantly, possibly occurring over 1-2 months [44] and requires decyanation, interaction of glutathione and in the case of methyl cobalamin, S-adenosyl methionine and the active form of folic acid. Qubeck study conducted on pigs showed that the intestinal absorption of vitamin B-12 from milk was higher than that of the synthetic form of vitamin B-12 by comparing the net fluxes of vitamin B-12 across the portal drained viscera after ingestion of milk or synthetic vitamin B-12 [45]. Vitamin B-12 in milk is in the form of coenzyme (adenosyl cobalamin), a major form in cellular tissues, where it is retained in the mitochondria, facilitating faster metabolic actions [46].

Even though the increase in plasma vitamin B-12 concentrations was modest (29.6 pmol/L) following milk intake by the participants, such an increase would be beneficial in vitamin B-12 deficient subjects. The increase in plasma holo-TC concentration after regular milk intake helped in lowering plasma tHcy concentrations (by 7 μmol/L).

The limitation of the present study is that we have not measured plasma methylmalonic acid concentration which is a specific biomarker for vitamin B-12 deficiency status. The other marker, tHcy gets accumulated in both vitamin B-12 and folate deficiency. However, none of our participants had folate deficiency. The participants with >148 pmol/L of plasma vitamin B-12 concentrations were not studied after 14 days consuming 400 ml milk every day.

The recommended dietary allowance (1 μg/day) for Indians seems to be inadequate. Even though there was significant reduction in plasma tHcy concentration 14 days after providing regular dietary vitamin B-12 (2.5 to 3 μg) in the form dairy products, the levels did not reach to normal levels. Effect of long term daily intake of milk on vitamin B-12 status should form a part of future study.

## Conclusions

In conclusion, the present study reveals that dairy foods provide a highly bio-available source of vitamin B-12. Plasma holo-TC concentration increase 24 hrs after milk load can be a marker for dietary vitamin B-12 absorption ability. Adequate milk intake has the potential to increase circulating vitamin B-12 and holo-TC concentrations and reduce tHcy concentrations, a risk factor for cardio-vascular disease. This study has implications for guidelines for the maintenance of normal vitamin B-12 status. Further study on large number of subjects with long term advice to consume adequate milk and observe the beneficial effects on health due to the improvement in vitamin B-12 status is needed. Then promotion of an adequate milk intake would be a good therapeutic strategy.

## Abbreviations

Holo-TC: Holotranscobalamin II; tHcy: Total homocysteine; mcv: Mean corpuscular volume.

## Competing interests

The authors declare that they have no competing interests.

## Authors’ contributions

Contributors: SSN was responsible for the study design, SSN, VB and AB had the primary responsibility to evaluate and write, NPM, SP and RT carried out the experimental work, the collection, analysis and statistical calculations regarding the blood tests. MK was responsible in statistical analysis. All authors read and approved the final manuscript.

## Authors’ information

Dr. Sadanand Naik: HOD (Clinical Biochemistry), Deenanath mangeshkar Hospital & Research Centre, Pune.

M.Sc. Ph.D. (Med. Biochemistry): Important contribution in Nutrition (1) One of the group members who coined the term Thin Fat Indian Baby (2) Original contribution in Early life nutritional exposure (intra uterine and neonatal) play a prominent role in programming the susceptibility to chronic diseases, such as obesity, cardio vascular disease, diabetes mellitus, and osteoporosis. (3) Adiposity and Insulin Resistance originate from birth (Dr. C.S.Yajnik and group).
